# Muscle Fascicles Exhibit Limited Passive Elongation Throughout the Rehabilitation of Achilles Tendon Rupture After Percutaneous Repair

**DOI:** 10.3389/fphys.2020.00746

**Published:** 2020-07-21

**Authors:** Alison N. Agres, Adamantios Arampatzis, Tobias Gehlen, Sebastian Manegold, Georg N. Duda

**Affiliations:** ^1^Julius Wolff Institute, Charité – Universitätsmedizin Berlin, Corporate Member of Freie Universität Berlin, Humboldt-Universität zu Berlin, and Berlin Institute of Health, Berlin, Germany; ^2^Department of Training and Movement Sciences, Humboldt University of Berlin, Berlin, Germany; ^3^Center for Musculoskeletal Surgery, Charité – Universitätsmedizin Berlin, Corporate Member of Freie Universität Berlin, Humboldt-Universität zu Berlin, and Berlin Institute of Health, Berlin, Germany; ^4^Department for Foot and Ankle Surgery, BG Unfallklinik Frankfurt am Main, Frankfurt am Main, Germany

**Keywords:** muscle-tendon unit, achilles tendon rupture, ultrasonography, ultrasound, rehabilitation, achilles tendon – injuries

## Abstract

Achilles tendon rupture (ATR) results in long-term functional and structural deficits, characterized by reduced ankle mobility and plantarflexor muscle atrophy. However, it remains unclear how such functional impairments develop after surgical repair. While it is known that this injury negatively affects the tendon’s function, to date, limited work has focused on the short-term effect of ATR on the structure of the muscles in series. The aim of this study was to characterize changes in medial gastrocnemius architecture and its response to passive lengthening during the post-surgical rehabilitative period following ATR. Both injured and contralateral limbs from 10 subjects (1 female, BMI: 27.2 ± 3.9 kg/m^2^; age: 46 ± 10 years) with acute, unilateral ATR were assessed at 8, 12, and 16 weeks after percutaneous surgical repair. To characterize the component tissues of the muscle-tendon unit, resting medial gastrocnemius muscle thickness, fascicle length, and pennation angle were determined from ultrasound images with the ankle in both maximal plantarflexion and dorsiflexion. The ankle range of motion (ROM) was determined using motion capture; combined ultrasound and motion capture determined the relative displacement of the musculotendinous junction (MTJ) of the AT with the medial gastrocnemius. The ATR-injured gastrocnemius muscle consistently exhibited lower thickness, regardless of time point and ankle angle. Maximal ankle plantarflexion angles and corresponding fascicle lengths were lower on the injured ankle compared to the contralateral throughout rehabilitation. When normalized to the overall ankle ROM, both injured fascicles and MTJ displacement exhibited a comparably lower change in length when the ankle was passively rotated. These results indicate that when both ankles are passively exposed to the same ROM following ATR surgery, both ipsilateral Achilles tendon and gastrocnemius muscle fascicles exhibit limited lengthening compared to the contralateral MTU tissues. This appears to be consistent throughout the rehabilitation of gait, suggesting that current post-operative rehabilitative exercises do not appear to induce muscle adaptations in the affected MTU.

## Introduction

Though the incidence of Achilles tendon rupture (ATR) has been steadily increasing (Lantto et al., 2014), its treatment remains unclear (Chiodo et al., 2010). Following injury, ATR patients exhibit lasting functional deficits in plantarflexor strength (Mullaney et al., 2006; Metz et al., 2009; Olsson et al., 2011; Agres et al., 2015; Heikkinen et al., 2017b) and in athletic performance (Amin et al., 2013; Trofa et al., 2017). Such deficits in function are likely due to lasting structural changes in both tissues of the affected plantarflexor muscle-tendon unit (MTU) (Heikkinen et al., 2017b), which presents with a longer tendon length (Kangas et al., 2007; Rosso et al., 2013; Agres et al., 2018) and triceps surae muscle atrophy, regardless of either surgical or conservative treatment (Rosso et al., 2013).

The introduction of loading on the MTU through rehabilitative exercise has been proposed as a means to induce adaptations in the constituent tissues immediately following injury. Such early interventions appear to have an impact on functional outcomes (Holm et al., 2015) and have been effective in improving MTU tissue properties, for example, in the tendons of healthy participants (Arampatzis et al., 2010; Bohm et al., 2014), and in the muscle architecture of elderly patients following hip surgery (Suetta et al., 2008). Weight bearing soon after initial ATR treatment has been seen as an ideal rehabilitative treatment for patients (Brumann et al., 2014). However, various forms of exercise and mobilization interventions in ATR rehabilitation do not appear to significantly improve functional outcomes (Suchak et al., 2008; Agres et al., 2018; Eliasson et al., 2018; Kastoft et al., 2019), particularly with regards to the recovery of plantarflexor strength on the affected limb. Considering the main contribution of the triceps surae muscle to plantarflexion performance, comparably limited work has investigated structural changes in the triceps surae muscles following ATR.

Adaptations in muscle generally occur at a much faster rate than those in tendon when exposed to the same loads (Mersmann et al., 2014). In particular, muscle remodeling following ATR injury is rapid compared to the rate of tendon healing, which can last as long as 1 year (Sharma and Maffulli, 2005). Side-to-side differences in muscle morphology are apparent soon after ATR, as Hullfish and colleagues have recently reported that medial gastrocnemius fascicles on the ATR-injured side remain both shorter and more pennate within 1 month post-injury (Hullfish et al., 2019). Recent findings suggest that further muscle remodeling occurs after this time, with increases in medial gastrocnemius volume found between 6 and 26 weeks after ATR (Eliasson et al., 2018). It remains unclear if immediate side-to-side differences in muscle architecture persist in later stages of rehabilitation, particularly when patients regain independent, unassisted gait.

During gait, the MTU and its constituent tissues undergo various length changes, which ultimately affect and determine their capacity for force production. In particular, medial gastrocnemius fascicles show an active shortening-stretch-shortening cycle during the stance phase of walking in healthy subjects, which suggest that sarcomeres can operate near its optimal length. During gait, the majority of the stance phase (about 15–75%) is dedicated to fascicle lengthening (Ishikawa et al., 2007). Once fascicle lengthening has peaked, the gastrocnemius muscle is then activated to provide plantarflexor force to the ankle.

As a result, the lengthening capacity of muscle fascicles plays a major role in force production and overall MTU function. Within the context of ATR, an increased AT length implies that the overall operating length of the muscle in series, and consequently also sarcomere length, is comparably shorter than uninjured MTUs (Suydam et al., 2015). This, combined with increased compliance in the healing tendon (Wang et al., 2013; Wulf et al., 2017; Agres et al., 2018), suggests that the triceps surae muscle fascicles on the ATR injured side are exposed to different length changes compared to healthy fascicles.

Thus, the primary aim of this study was to determine the effect of passive sagittal ankle motion on relevant structures of the post-ATR MTU, namely muscle architecture parameters and AT length, during the mid-to-late stages of rehabilitation. To achieve this, the injured and contralateral structures of both muscle and tendon in ATR patients were assessed in maximal plantarflexion and dorsiflexion at 8, 12, and 16 weeks post-surgery. We hypothesized that throughout the rehabilitative period, the muscle fascicles on the ATR-injured MTU would demonstrate lower fascicle elongation when exposed to passive lengthening compared to the contralateral side. Furthermore, we hypothesized that this would be accompanied by a decreased AT elongation on the ATR-injured leg, as determined by displacements of the musculotendinous junction (MTJ). Finally, we postulated that when exposed to the same passive motion, relative changes in muscle thickness and pennation angle on the ATR-injured muscle would be lower than compared to the contralateral side.

## Materials and Methods

### Patients

**FIGURE 1:**
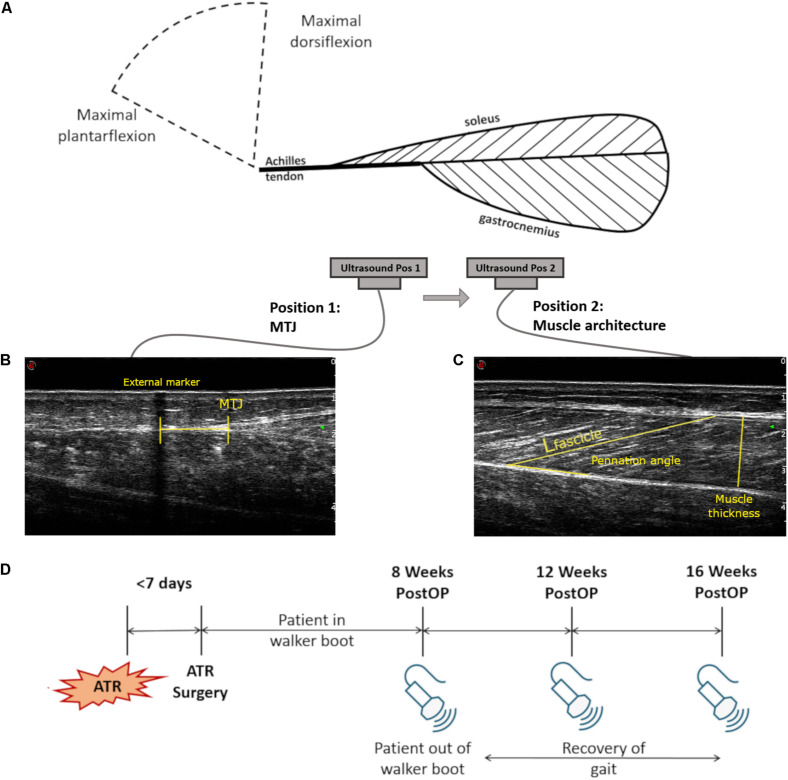
Measurement setup and study description. **(A)** Schematic of the measurement setup for the ultrasound capture, showing the muscle-tendon unit (MTU) tissues and the two positions of the ankle and the two relative positions of the single ultrasound probe. Two separate ultrasound images were taken at both maximal plantarflexion and dorsiflexion: **(B)** Sample ultrasound image locating the musculotendinous junction (MTJ) displacement of the Achilles tendon to the medial gastrocnemius with respect to an external skin marker. **(C)** Sample ultrasound image of medial gastrocnemius architecture measures at the midbelly of the calf, illustrating how fascicle length (*L*_fascicle_), muscle thickness, and pennation angle were assessed. **(D)** Timeline of the three measurement sessions with relation to the time of ATR injury and surgical treatment.

The present work was part of a larger experimental prospective study that aimed to determine the effect of a specific percutaneous surgical repair of ATR on overall function. A subset of 10 participants (46 ± 10 years old; BMI: 27.2 ± 3.9 kg/m^2^; 1 female) were prospectively recruited after receiving percutaneous surgical repair using the Dresden instrument (Amlang et al., 2006) by a single surgeon within 7 days of initial injury. This cohort was assessed at 8, 12, and 16 weeks after surgical treatment of an acute, unilateral ATR (Figure 1D). Patients were included if classified as a Type 2a or 2b rupture after clinical ultrasound assessment of the initial injury (Amlang et al., 2011). Exclusion criteria included immobilized patients, open ATR surgery, non-surgical ATR treatment, concomitant injury to either the ipsilateral or contralateral lower extremity within 6 months of the ATR, re-rupture of the AT, and contralateral ATR. Patients received percutaneous surgery within 1 week of ATR injury. All patients gave informed consent prior to participation. The local ethics committee approved this study (EA/2/095/11) and all protocols were developed in accordance with the Declaration of Helsinki.

Due to technical problems with one data set at the 8-week follow-up, there was only a total of 9 patient data sets available for analysis for the initial time point. Furthermore, due to two drop-outs for the last time point, only 8 data sets were available for the 16-week follow-up.

### Measurement Setup

A set of seven infrared motion capture cameras (MX F20, *f* = 250 Hz, VICON, Oxford, United Kingdom) was used to determine the ankle angles from each limb. Markers were placed on the medial and lateral condyles of the femur, medial and lateral malleoli, the 1st head of the metatarsal, the calcaneus, the lateral aspect of the thigh, and the greater trochanter. These marker positions were used to determine the ankle angle relative to a reference position, whereby the foot was in a neutral position, where motion capture data was collected. Simultaneous non-invasive assessment of the medial gastrocnemius muscle architecture and medial gastrocnemius MTJ was performed using a 10 cm, 7.5 MHz B-mode ultrasonography probe (*f* = 25 Hz, MyLab60, Esaote S.p.A., Genoa, Italy) while patients were seated with the knee outstretched and the ankle placed on a dynamometer pedal. Each ankle then was slowly brought to maximal plantarflexion and dorsiflexion, based on subjective feedback from the participant as to when pain or mobility limits were reached. The pedal was then held in place at this angle and paused. Once each of these positions was reached, both motion capture data and two ultrasound images were collected and synchronized (Figure 1A).

The first ultrasound image recorded medial gastrocnemius (GM) architectural parameters at the midbelly of the medial gastrocnemius (Figure 1B). The second image recorded the location of the musculotendinous junction (MTJ) of the insertion of the medial gastrocnemius into the Achilles tendon. For the second image, the relative displacement of the MTJ was determined by calculating the MTJ distance between plantarflexion and dorsiflexion, with reference to a small strip of tape directly placed on the skin. This functioned as an externally placed echo-absorbing skin marker, which remained unmoved for both measurement positions (Figure 1C). Furthermore, the rest length of the Achilles tendon on both sides was determined from the MTJ to the calcaneus with the patient in 20° of plantarflexion and an outstretched knee, due to the slack in the muscle-tendon unit at this position (De Monte et al., 2006).

### Image Analysis

Following collection of all images, the fascicle length, pennation angle, and thickness of the muscle were measured using open-source software (ImageJ 1.48v, National Institutes of Health, Bethesda, MD, United States) and a stylus-equipped monitor (Wacom Cintiq 21UX DTK-2100, Portland, OR, United States). Fascicle length was defined as the linear distance between aponeurosis insertions. Pennation angle was measured between the deep aponeurosis and the fascicle. Muscle thickness was measured as the shortest linear distance between the upper and lower aponeuroses (Figure 1B). Due to variations in muscle architecture along its length, three measurements of each parameter were measured and averaged for each captured image. To avoid bias, images were randomized and blinded for the injured side during analysis.

To account for possible side-to-side differences in ankle sagittal plane mobility, all parameters gathered from image analysis were also normalized to the ankle range of motion (ROM). To describe this in detail: for a given parameter, *p*, the value at plantarflexion was subtracted from the corresponding value at dorsiflexion, yielding the absolute change of parameter, or Δ*p*. This quantity was calculated for each leg type, or side (Δ*p*_side_), and then normalized to the ROM corresponding side (ROM_side_). This results in ROM-normalized parameters, as described by the formula

$$\frac{\Delta p_{\text{side}}}{{\text{ROM}}_{\text{side}}}=\frac{p_{\mathrm{side}\left(\mathrm{dorsiflexion}\right)}-p_{\mathrm{side}\left(\mathrm{plantarflexion}\right)}}{\theta_{\mathrm{side}\left(\mathrm{dorsiflexion}\right)}-\theta_{\mathrm{side}\left(\mathrm{plantarflexion}\right)}}$$

where *p* refers to the parameter calculated (fascicle length, pennation angle, or muscle thickness), *side* refers to the limb of interest (ATR injured or contralateral), and θ refers to the ankle angle in degrees.

### Statistics

Means, standard deviations, and 95% confidence intervals were calculated for all parameters from each patient at each time point. All parameters were tested for normality using Shapiro-Wilk, then for sphericity using Mauchly’s test in SPSS (IBM, Armonk, NY, United States). A factorial repeated-measures analysis of variance (ANOVA) was performed to analyze differences with consideration of two main effects: the time of measurement post-surgery (8, 12, or 16 weeks) and the leg type (ATR injured or contralateral). If sphericity could not be assumed according to Mauchly’s test, a Greenhouse-Geisser correction was applied to the factorial repeated-measures ANOVA. A Bonferroni adjustment was applied to the original level of significance (α = 0.05) due to the multiple comparisons performed. Thus, the final level of significance was set at α = 0.01 (α = 0.05/5). When significant contrasts were found, the effect size was calculated for the relevant pair and interpreted according to Cohen (1988).

In order to determine the intra-rater reliability of ultrasound image processing, an experienced investigator blinded to the patient type and injured side assessed all parameters for the dorsiflexion position in two separate analysis sessions. A two-way mixed-effects intraclass correlation coefficient (ICC) for absolute agreement in single measures (McGraw and Wong, 1996) was calculated for each of the three measured parameters.

## Results

The absolute values of all parameters are listed in Table 1 for the included subjects where data was available for all time points (*n* = 8) for reference. There was no significant effect of time of measurement post-surgery on any of the investigated parameters. Thus, the results below detail findings from the factorial repeated-measures ANOVA with regards to the main effect of leg type, as well as the interaction of time of measurement with leg type. These are presented with means, significance values, effect sizes (for significant findings), and 95% confidence intervals for the difference of the ATR-injured side (INJ) from the contralateral side (CON).

**TABLE 1:** Absolute values of ankle angles and muscle architecture parameters on the ATR injured and contralateral (CON) musle-tendon units for included subjects (*n* = 8) at two ankle positions at three different measurement time points during the rehabilitation period.

		Ankle position			
		Maximum plantarflexion		Maximum dorsiflexion	
Parameter	Time post-op	ATR	CON	ATR	CON
Ankle angle (°)	8 weeks	26.8 ± 5.9	31.9 ± 2.7	−4.9 ± 6.0	−8.1 ± 5.2
	12 weeks	29.3 ± 7.2	37.1 ± 5.8	−7.8 ± 8.0	−4.3 ± 6.5
	16 weeks	26.8 ± 3.7	35.4 ± 5.1	−10.0 ± 2.9	−4.4 ± 5.0

Fascicle length (mm)	8 weeks	38.1 ± 2.3	39.4 ± 4.1	49.0 ± 4.7	57.7 ± 4.0
	12 weeks	36.5 ± 5.9	46.0 ± 12	45.7 ± 5.8	59.9 ± 10.3
	16 weeks	35.6 ± 5.3	40.1 ± 7.6	41.9 ± 6.8	56.7 ± 7.9

Muscle thickness (mm)	8 weeks	17.9 ± 2.9	20.3 ± 2.7	15.4 ± 2.6	18.4 ± 2.2
	12 weeks	17.0 ± 2.4	20.7 ± 3.0	15.7 ± 3.0	18.6 ± 3.6
	16 weeks	16.8 ± 3.1	20.8 ± 3.5	15.2 ± 3.7	18.3 ± 3.5

Pennation angle (°)	8 weeks	22.0 ± 3.3	27.4 ± 3.5	19.3 ± 1.3	19.3 ± 2.2
	12 weeks	26.2 ± 6.8	24.8 ± 6.0	22.4 ± 5.9	19.9 ± 3.8
	16 weeks	28.6 ± 7.6	28.3 ± 5.9	22.3 ± 5.3	20.2 ± 2.5

*Values are reported as means ± SD of the average values for all subjects. Ankle joint angle is defined with reference to a neutral ankle position set at 0°, where positive values indicate plantarflexion and negative values indicate dorsiflexion.*

### Rest Tendon Length

The Achilles tendon length at 20 degrees of plantarflexion was significantly longer on INJ compared to CON (20.6 vs 18.6 cm, *p* = 0.004, CI [3.0 to 35.7] cm) during the rehabilitation period. However, no interaction effects were found between the main effects of leg type and time point.

### Parameters at Maximal Plantarflexion

**FIGURE 2:**
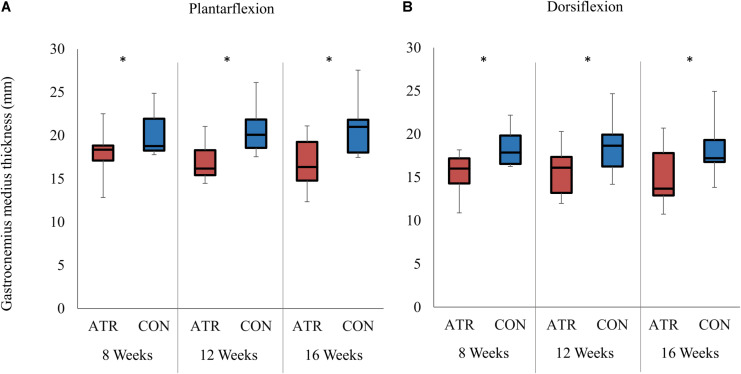
Medial gastrocnemius muscle thickness on the ATR-injured and contralateral limbs at maximal plantarflexion **(A)** and dorsiflexion **(B)**. Significant levels for the overall effect of leg type as determined by a factorial repeated-measures ANOVA (*p* < 0.01) are indicated with *.

The measured ankle angle at maximal plantarflexion was found to be significantly lower on INJ compared to CON (Table 1, 26.8 vs 35.1°, *p* = 0.002, *r* = 0.91, CI [−12.0 to −4.5]°). When the foot was held in maximal plantarflexion, there was a significant main effect of leg type on medial gastrocnemius thickness (Figure 2A, 17.3 vs 20.6 mm, *p* = 0.002, *r* = 0.90, CI [−4.8 to −1.8] mm) and on muscle fascicle length (38.2 vs 42.0 mm, *p* = 0.023, CI [−8.1 to 0.55] mm). Both parameters were lower on on INJ compared to CON. Pennation angle (25.5 vs 26.9°, *p* = 0.136, CI [−3.55 to 0.62] °) did not exhibit any significant effects of leg type at maximal plantarflexion. Furthermore, no interaction effects were found between time of measurement and leg type at maximal plantarflexion.

### Parameters at Maximal Dorsiflexion

The measured ankle angle at maximal dorsiflexion did not exhibit any effects of leg type at maximal dorsiflexion (Table 1, −8.0 vs −5.6°, *p* = 0.102, CI [−5.39 to 0.64] °). At this dorsiflexed angle, there was a significant main effect of leg type on medial gastrocnemius thickness (Figure 2B, 15.4 vs 18.4 mm, *p* = 0.002, *r* = 0.90, CI [−4.5 to −1.5] mm), with INJ values lower than CON. Similarly, muscle fascicle length at maximal dorsiflexion also exhibited a significant main effect of leg type (45.5 vs 58.1 mm, *p* < 0.001, *r* = 0.98, CI [−15.0 to −10.1] mm). However, pennation angle (21.3 vs 19.9°, *p* = 0.188, CI [−0.92 to 3.76] °) did not exhibit any significant effects of leg type at maximal dorsiflexion. Furthermore, no interaction effects were found between time of measurement and leg type at maximal dorsiflexion.

### Absolute Changes of Parameters in the Range of Motion

**FIGURE 3:**
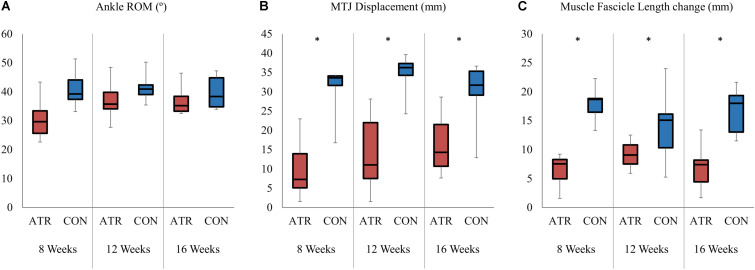
Absolute changes of selected parameters between maximal plantarflexion and maximal dorsiflexion for both the on the ATR-injured and contralateral (CON) sides at each measurement point. Significant levels for the overall effect of leg type as determined by a factorial repeated-measures ANOVA (*p* < 0.01) are indicated with *. **(A)** Range of motion of the ankle (°). **(B)** Absolute displacement of the musculotendinous junction (MTJ, in mm). **(C)** Absolute change in the medial gastrocnemius fascicle length (mm).

Ankle mobility, as determined by the ankle ROM, was found to be similar between INJ and CON, with no effect of leg type (34.8 vs 40.7°, *p* = 0.033, CI [−11.2 to 0.66] °, Figure 3A). However, the absolute change in relative MTJ displacement exhibited a significant main effect of leg type (*p* = 0.001, *r* = 0.94), with INJ displacements lower than CON (Figure 3B). Absolute changes in muscle fascicle length similarly exhibited a significant main effect of leg type (8.8 vs 16.2 mm, *p* < 0.001, *r* = 0.95, CI [−9.9 to −5.0] mm, Figure 3C). Between maximal plantarflexion and maximal dorsiflexion, the change of muscle fascicle length on INJ was significantly lower than on CON. Both overall changes in medial gastrocnemius thickness (1.8 vs 2.1 mm, *p* = 0.496, CI [−1.3 to 0.7] mm) and pennation angle (−4.2 vs −7.0°, *p* = 0.039, CI [0.2 to 5.4] °) did not exhibit any significant effects of leg type.

There was a significant interaction between time of measurement and leg type for ankle ROM (*p* = 0.003). Contrasts were performed and revealed significant interactions when comparing INJ to CON for the 8 weeks time point and the 16 weeks time point (*p* = 0.013, *r* = 0.83), with INJ ROM increasing more with time compared to CON. The remaining contrast revealed no significant interaction term when comparing INJ to CON for the 12 weeks and the 16 weeks time point (*p* = 0.505, *r* = 0.28).

### Normalized Changes of Parameters to the Range of Motion

**FIGURE 4:**
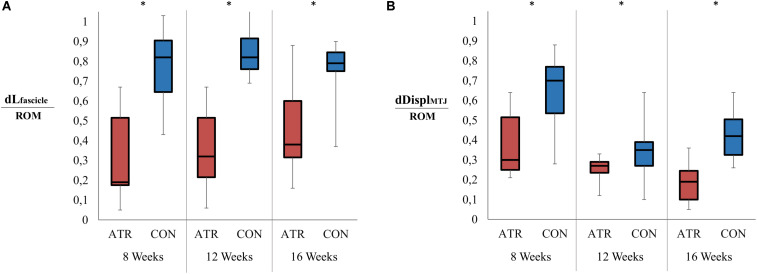
Ranges in relevant muscle-tendon unit architecture parameters between maximal plantarflexion and maximal dorsiflexion, normalized to the overall ankle range of motion (ROM). Significant levels for the effect of leg type [as determined by a factorial repeated-measures ANOVA (*p* < 0.01)] are indicated with *. **(A)** Relative change in the medial gastrocnemius fascicle length (mm) as normalized to the ankle range of motion (°). **(B)** Displacement of the musculotendinous junction (MTJ, in mm) as normalized to the ankle range of motion (°).

Overall changes in muscle fascicle length, when normalized to overall ankle ROM, displayed a significant main effect of leg type (0.27 vs 0.47 mm/°, *p* = 0.001, *r* = 0.93, CI [−0.28 to −0.13] mm/°), with relative changes of INJ muscle fascicle length lower than CON (Figure 4A). Furthermore, overall MTJ displacement normalized to total ankle ROM also exhibited a significant main of leg type (0.38 vs 0.79 mm/°, *p* = 0.001, *r* = 0.92, CI [−0.58 to −0.23] mm/°) (Figure 4B). This ROM-normalized MTJ displacement on INJ was found to be lower than that of CON.

When the overall change in medial gastrocnemius thickness was normalized to ankle ROM, no effect of leg type was found (0.056 vs 0.053 mm/°, *p* = 0.843, CI [−0.031 to 0.037] mm/°). Similarly, ROM-normalized changes in pennation angle were found to be similar between INJ and CON sides (−0.13 vs −0.18, *p* = 0.199, CI [−0.037 to 0.14]). For all examined parameters, there was no interaction found between the main effects of the time of measurement post-surgery and leg type.

### Image Analysis Reliability

Intra-rater reliability was found to be excellent for fascicle length (ICC = 0.955) and very good for pennation angle (ICC = 0.840) and MTJ displacement (ICC = 0.844). The intra-rater reliability of muscle thickness was good with an ICC value of 0.757.

## Discussion

**FIGURE 5:**
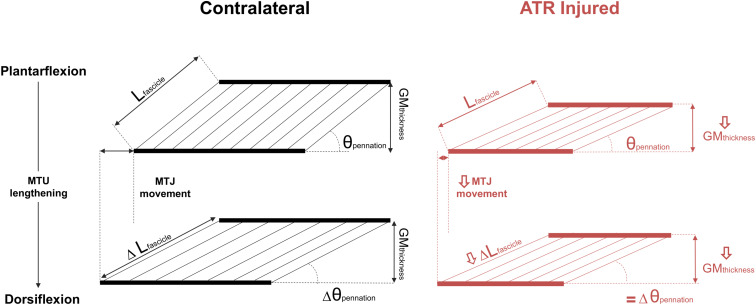
A schematic summarizing the observed differences in MTU tissues when exposed to passive MTU lengthening between the contralateral (CON, left column, black) and ATR-injured tissues (right column, red) after surgical ATR repair. The top row depicts the two muscles as seen in plantarflexion, when the MTU is comparably slack, and the bottom row depicts the two muscles as seen in dorsiflexion, when the MTU is held in more tension. Absolute differences are indicated in the bottom row with Δ ahead of the parameter, as the difference between plantarflexion and dorsiflexion. The top left diagram depicts the parameters of interest on the contralateral side: *L*_fascicle_ is the measured length of the medial gastrocnemius fascicle, θ_pennation_ denotes the pennation angle, GM_thickness_ denotes the measured thickness of the medial gastrocnemius, and the distance between the dashed lines indicates the relative movement of the musculotendinous junction (MTJ). Arrows on the right column ATR parameters indicate significant differences to CON at the same position. Equal signs indicate no significant differences compared to CON.

This longitudinal study determined the effect of passive ankle motion on the architecture and structure of the component tissues of the MTU during the rehabilitative period after percutaneous surgical repair of an ATR, when compared to the uninjured contralateral side. Taking previous reports of increased AT length and compliance into consideration, it was hypothesized that the medial gastrocnemius muscle would exhibit lower fascicle elongation when exposed to passive lengthening, together with limited MTJ displacement. The results here support this hypothesis, indicating that when normalized to the available ROM in the ankle, both the tendon and the muscle fascicles consistently exhibit a lower capacity to passively lengthen on the ATR side when compared to the contralateral side post-surgery. However, the hypothesis postulating that relative changes in muscle thickness and pennation angle would be lower on the ATR-injured side were not supported within this work. These results indicate that when both ankles are passively exposed to the same ROM following ATR surgery, both ipsilateral Achilles tendon and gastrocnemius muscle fascicles exhibit limited lengthening compared to the contralateral MTU tissues (Figure 5). This appears to be consistent throughout the rehabilitation of gait in this particular cohort.

These findings imply that restoration of side-to-side passive ankle ROM, a common clinical benchmark, does not reflect the remaining side-to-side differences in passive fascicle lengthening following ATR surgery. Such limitations in fascicle lengthening on the ATR side may have multiscale implications on the affected muscle in parallel. Recent work investigating the effect of passive lengthening on gastrocnemius architecture in healthy subjects has determined that changes in fascicle length are comparable to length changes in muscle belly length (Bolsterlee et al., 2017). Passive changes in fascicle length may also be indicative of changes at the sarcomere level, as observed in multiscale *in vivo* investigations of passive fascicle and sarcomere length in the tibialis anterior muscle (Lichtwark et al., 2018). Based on the observations at the fascicle level within this study, passive sarcomere-level length changes may also be limited on the ATR side compared to the contralateral side. Overall, this suggests that following ATR surgery, the sarcomeres on the ATR side operate on a different portion of the length-tension relationship compared to the contralateral side, even if there are no side-to-side differences in ankle ROM.

Throughout the rehabilitation period, ATR muscle fascicles consistently exhibited a shorter length through the available ROM compared to the contralateral side (Figure 3C). This was accompanied by limited MTJ displacement throughout the same ROM (Figure 3B). These results are comparable to previous short-term measurements that found consistently shorter fascicle lengths and lower muscle thickness up to 4 weeks post-ATR when held in plantarflexion (Hullfish et al., 2019). This appears to be consistent throughout the rehabilitation of gait, suggesting that current post-operative rehabilitative exercise do not appear to induce muscle adaptations on fascicle length in the affected MTU.

The results within this work suggest that limited to no muscle hypertrophy occurs in the medial gastrocnemius during rehabilitation between 8 and 16 weeks. This is in contrast with findings from other work. In particular, pennation angle was not observed to significantly change with time within this study, which contrasts short-term findings from Hullfish and colleagues, who found significant increases within 1 month post-ATR. Surprisingly, within the time frame investigated, muscle fascicle length did not significantly change, which explains no changes in muscle thickness on the injured muscle during the rehabilitative period. These results are in contrast with other follow-ups of volumetric changes in these muscles in ATR patients, which found that the medial gastrocnemius significantly increased in cross-sectional area between 6 and 26 weeks post-ATR (Eliasson et al., 2018). A possible explanation for this may lie in the method that muscle was assessed. There are considerable limitations in the two-dimensional assessment of muscle using ultrasound, particularly since transducer position can affect measures such as fascicle length (Bolsterlee et al., 2016). Though recent work suggests that there is a correlation between two-dimensional muscle thickness and MRI-measured cross-sectional area in athletes (Franchi et al., 2018), this may not hold true within the context of pathologies such as ATR.

The results here indicate that during the mid- to late-stages of rehabilitation after ATR surgery, when patients are removed from assistive devices and begin to ambulate independently, that adaptive behavior in the medial gastrocnemius muscle on the ATR side is limited compared to the uninjured contralateral side. This is in stark contrast to what was observed in the patients themselves in day-to-day activities like gait, as all subjects were fully mobile in daily activities by 16 weeks. This mismatch between the lack of difference in overall ankle mobility and sustained physiological deficits at the MTU-level further support the theory that compensatory action by neighboring muscle groups, such as the flexor hallucis longus (Finni et al., 2006; Heikkinen et al., 2017a), may be ultimately responsible for the recovery of the ATR patient. However, these compensatory actions are unable to restore previous function, particularly in high-level sports (Amin et al., 2013). This suggests that current rehabilitative exercise measures performed within this period of ATR rehabilitation (specifically, recovery of independent gait) may not induce adaptations in the targeted MTU muscles, but rather allow for the development of compensatory adaptations for disadvantages in the affected triceps surae muscle.

These results further imply that a combination of increased rest tendon length and increased tendon compliance following surgical ATR treatment negatively affects passive force transmission between the two constituent tissues of the muscle-tendon unit. This directly impacts the efficacy of active force transmission from the ATR-side muscle to the tendon in order to actively plantarflex the ankle. To potentially compensate for these side-to-side morphological differences, contractile force in the gastrocnemius could be increased by augmenting the activation of the muscle itself. Longer-term follow ups in ATR patients indicate that both the lateral and medial gastrocnemius exhibit higher integrated EMG activity on the ATR side compared to the contralateral side during gait, as found in patients that similarly exhibited longer Achilles tendon rest length on the ATR side (Suydam et al., 2015). Yet despite these modulations at the neuromuscular level, they ultimately cannot overcome the negative morphological changes in the muscle. Here, the ATR-affected gastrocnemius appears to both have a shorter overall length within the MTU and lower thickness compared to the contralateral limb, suggesting overall volumetric atrophy and further reducing the MTU’s capacity for active plantarflexion. The findings presented here, when placed in context of previous findings, suggest that negative adaptations in the morphology of both MTU constituent tissues following ATR aggregate, ultimately leading to limited passive force transmission between the muscle and tendon, which in turn limits the MTU’s capacity to actively plantarflex the ankle.

A limitation of this work is that only the AT was considered within the context of the tendinous structures, and no investigations of the aponeuroses were performed. These results do not exclude the possibility that changes in elasticity could also affect the deep and superior aponeuroses of the gastrocnemius muscle. Increased lengthening of the entire MTU leads to limited deformation in the Achilles tendon in series, but this does not exclude the possibility that components of the parallel elastic component have also become more compliant. Another limitation is that for all measured positions, the knee remained outstretched and, thus, the plantarflexed position may not represent a perfectly slack MTU rest position, with the knee flexed and the ankle neutral, both at 90° (Grieve et al., 1978). However, slackness in the MTU has been shown to be present at similar knee and ankle angles as was measured here (De Monte et al., 2006). Thus, we are confident in our measurement positions of the MTU for the plantarflexed position as representative of a slack MTU. A further limitation of this study is the subjective method used to assessed the limits of ankle ROM, which was determined based on the subject’s self-reported pain and discomfort while seated in the dynamometer. This method was chosen for the safety and security of the patient during measurement sessions. As pain thresholds vary from subject to subject, this introduces variability in the ROM measurements. However, considering most reports of passive ROM often use manual goniometers to perform measurements, we feel more confident in our use of motion capture to determine this parameter.

A final limitation of this study is the lack of a healthy control comparison group to compare to both limbs of the ATR injured group, which were not available within the context of this initial study. Previous existing studies in healthy participants have already extensively investigated how passive changes in ankle angle affect muscle fascicle length at different locations of the muscle belly using multiple imaging modalities (Herbert et al., 2002, 2011; Bolsterlee et al., 2017). The results from this work, however, give a first indication that within-subject differences are present, even within this small cohort. Future studies should aim to include matched controls to ATR patients, to determine if adaptations on both INJ and CON muscle fascicles are distinct from those in uninjured, healthy participants.

## Conclusion

This study shows that following ATR surgery and during rehabilitation, the muscle fascicles of the affected MTU exhibit a limited capacity to lengthen when the ankle is passively moved into dorsiflexion when compared to the contralateral side. This is coincident with limited passive AT lengthening on the affected MTU, as measured by MTJ displacement. Furthermore, these altered passive properties are accompanied by sustained medial gastrocnemius atrophy, as measured by muscle belly thickness, as well as a longer Achilles tendon, as measured at rest. These collective results indicate that both tendon and muscle alter their passive structural properties soon after ATR and remain this way, even during the introduction of more rehabilitative exercise and movement. Furthermore, when both ankles are passively exposed to the same ROM following ATR surgery, both ipsilateral Achilles tendon and medial gastrocnemius muscle fascicles exhibit limited lengthening compared to the contralateral MTU tissues. Our findings indicate that current rehabilitative exercises that aim to improve plantarflexor muscle strength may not effectively target the muscle tissues of the affected MTU in the short- to mid-term following surgical repair of an ATR. This appears to be consistent throughout the rehabilitative period, suggesting that current post-operative rehabilitative exercise do not appear to induce muscle adaptations in the affected MTU following surgery. The results here imply that impaired passive force transmission on the ATR side likely stems from negative morphological adaptations in both the affected tendon and the muscle tissues. Further investigation of rehabilitative exercise interventions within the context of muscle adaptation after surgical ATR repair are warranted.

## Data Availability Statement

The datasets generated for this study are available on request to the corresponding author.

## Ethics Statement

All study protocols were reviewed and approved by Ethikkommission der Charité-Universitätsmedizin Berlin. The patients provided their written informed consent to participate in this study.

## Author Contributions

ANA, AA, SM, and GD designed the experimental study. SM performed surgeries. SM and TG recruited participants. ANA and TG performed the data collection. ANA, AA, and GD interpreted the results and drafted the manuscript. SM and TG contributed to the revision of the text. All authors contributed to the article and approved the submitted version.

## Conflict of Interest

The authors declare that the research was conducted in the absence of any commercial or financial relationships that could be construed as a potential conflict of interest.
